# Angiogenin Levels and *ANG* Genotypes: Dysregulation in Amyotrophic Lateral Sclerosis

**DOI:** 10.1371/journal.pone.0015402

**Published:** 2010-11-10

**Authors:** Russell Lewis McLaughlin, Julie Phukan, William McCormack, David S. Lynch, Matthew Greenway, Simon Cronin, Jean Saunders, Agnieska Slowik, Barbara Tomik, Peter M. Andersen, Daniel G. Bradley, Phil Jakeman, Orla Hardiman

**Affiliations:** 1 Smurfit Institute of Genetics, Trinity College Dublin, Dublin, Ireland; 2 Department of Neurology, Beaumont Hospital, Dublin, Ireland; 3 Trinity College Institute of Neuroscience, Trinity College Dublin, Dublin, Ireland; 4 Human Science Research Unit, Faculty of Education and Health Sciences, University of Limerick, Limerick, Ireland; 5 Royal College of Surgeons Ireland, Dublin, Ireland; 6 Statistical Consulting Unit, University of Limerick, Limerick, Ireland; 7 Department of Neurology, Jagiellonian University, Krakow, Poland; 8 Department of Pharmacology and Clinical Neurosciences, Umeå University Hospital, Umeå, Sweden; Aarhus University, Denmark

## Abstract

**Objective:**

To determine whether 5 single nucleotide polymorphisms (SNPs) associate with ALS in 3 different populations. We also assessed the contribution of genotype to angiogenin levels in plasma and CSF.

**Methods:**

Allelic association statistics were calculated for polymorphisms in the *ANG* gene in 859 patients and 1047 controls from Sweden, Ireland and Poland. Plasma, serum and CSF angiogenin levels were quantified and stratified according to genotypes across the *ANG* gene. The contribution of SNP genotypes to variance in circulating angiogenin levels was estimated in patients and controls.

**Results:**

All SNPs showed association with ALS in the Irish group. The SNP rs17114699 replicated in the Swedish cohort. No SNP associated in the Polish cohort. Age- and sex-corrected circulating angiogenin levels were significantly lower in patients than in controls (p<0.001). An allele dose-dependent regulation of angiogenin levels was observed in controls. This regulation was attenuated in the ALS cohort. A significant positive correlation between CSF plasma angiogenin levels was present in controls and abolished in ALS.

**Conclusions:**

*ANG* variants associate with ALS in the Irish and Swedish populations, but not in the Polish. There is evidence of dysregulation of angiogenin expression in plasma and CSF in sporadic ALS. Angiogenin expression is likely to be important in the pathogenesis of ALS.

## Introduction

Angiogenin is the 14.1-kDa product of the hypoxia responsive gene *ANG* on chromosome 14. We have shown previously that mutations in *ANG* are associated with amyotrophic lateral sclerosis (ALS), and that *ANG* mutations predict loss of RNAse and angiogenic function [1]. Moreover, recent studies have suggested that angiogenin is an important neurodevelopmental protein with neuroprotective properties, and that mutant *ANG* impairs neurite outgrowth. [2]–[4].

Angiogenin is functionally similar to vascular endothelial growth factor (VEGF), altered regulation of which has also been associated with ALS [5], [6]. ‘At risk’ promoter haplotypes in VEGF, which predict reduced expression of bioavailable isoforms, have been described in some European ALS populations [7] and combined with evidence from animal models, the data suggest that VEGF isoforms have a neuromodulatory and neuroprotective role in the CNS. Despite the functional similarity between angiogenin and VEGF, there have been few studies to date that have investigated angiogenin expression and regulation in ALS.

We have recently shown that serum angiogenin levels in ALS differ from controls [8]. The patterns of plasma and cerebrospinal fluid (CSF) angiogenin expression have not previously been investigated, and there have been no studies to determine whether *ANG* haplotypes modulate protein expression, as is the case with VEGF. We have sought to determine (i) whether angiogenin is detectable in CSF, (ii) whether there is a consistent relationship between plasma and CSF angiogenin levels, (iii) whether genetic variations in the *ANG* locus control angiogenin expression, and (iv) whether, as has been reported for VEGF [9]–[11], there is a dysregulation of angiogenin in sporadic ALS.

## Methods

### Participants

**Figure 1:**
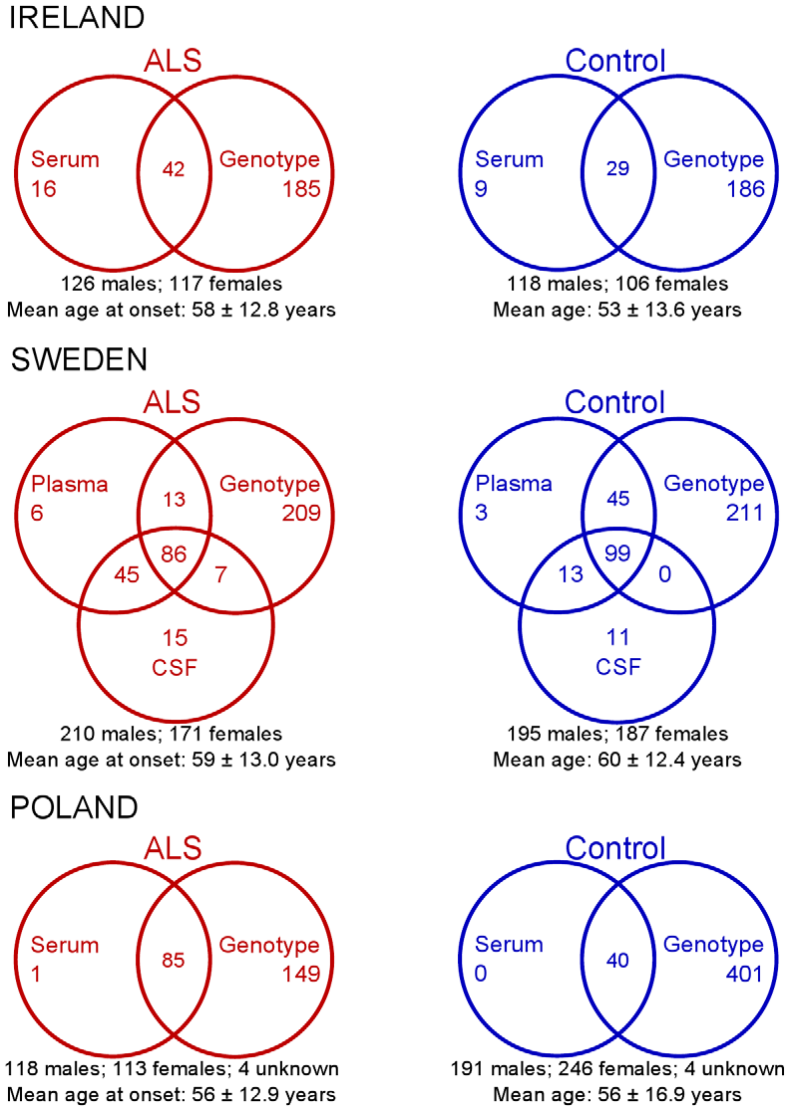
Numbers of individuals and demographics of the three study populations. Error values for mean ages represent standard deviation.

DNA and serum samples were drawn from Irish and Polish ALS patients; DNA, plasma and cerebrospinal fluid (CSF) samples were drawn from Swedish ALS patients. Unrelated control subjects with no family history of ALS were sampled from the same populations. The numbers of participants available in the three study populations and their demographics are detailed in figure 1. All patients fulfilled the El Escorial criteria for clinically definite or probable ALS [12]. Patients with atypical phenotypes and Swedish patients with mutations in the *SOD1* gene were excluded. Informed written consent was obtained from all participants and the study was approved by the ethics committees in Beaumont Hospital, Umeå University and the Jagelonian Institute.

### SNP genotyping

Using data from the CEPH panel of the International HapMap Project [13], 5 informative haplotype-tagging single nucleotide polymorphisms (htSNPs) were selected covering the *ANG* gene with inter-marker r^2^ below 0.8 and minor allele frequency above 5%. These htSNPs are detailed in table 1. Genotyping across these five htSNPs was performed commercially by KBiosciences (Herts, UK) using KASPar assays with standard quality-control criteria (genotypes formed three distinct clusters, water controls were negative and minor allele frequency was above 5%).

**Table 1:** Allele frequencies and SNP association statistics in the three populations.

SNP	Alleles	IRELAND				SWEDEN				POLAND			
		RA	RA freq	Allelic association		RA	RA freq	Allelic association		RA	RA freq	Allelic association	
			ALS;ctrl	p	OR		ALS;ctrl	p	OR		ALS;ctrl	p	OR
rs9322855	A>C	C	0.50; 0.59	0.003*	1.57	A	0.56; 0.52	0.13	0.85	A	0.55; 0.55	0.92	0.99
rs8004382	G>A	G	0.57; 0.47	0.007*	1.50	G	0.55; 0.52	0.46	0.92	G	0.55; 0.52	0.46	0.92
rs4470055	G>A	A	0.29; 0.22	0.03*	1.47	A	0.25; 0.24	0.66	1.06	G	0.75; 0.72	0.36	0.88
rs17114699	G>T	T	0.16; 0.11	0.03*	1.53	T	0.14; 0.08	0.001*	1.78	G	0.89; 0.87	0.68	0.93
rs11701	T>G	G	0.18; 0.10	0.006*	1.88	G	0.13; 0.13	0.69	1.07	G	0.13; 0.10	0.14	1.3

RA, risk allele; OR, odds ratio.

*Significant p-value.

### Quantification of angiogenin in CSF, plasma and serum

Serum and plasma were isolated from peripheral blood according to standard protocols. Since angiogenin has not been shown to have any interaction partners in the blood, plasma and serum angiogenin concentrations were considered to be comparable. Samples were stored at −80°C until assay. Angiogenin concentration was measured by enzyme-linked immunosorbent assay (ELISA) according to manufacturer's guidelines (Quantikine Duoset, R&D Systems, Abingdon, UK). All samples were assayed in duplicate and calibrated against serially diluted standards of known mass. Pooled CSF and plasma quality control (QC) samples were both assayed in duplicate on each mitrotitre plate, setting the precision of the assay across all microtitre plates. An inter-assay coefficient of variation (CV) of 6% and 8% was obtained for the high and low plasma QC respectively. An inter-assay CV of 9% was obtained for the CSF QC.

### Statistical analysis

Unless otherwise stated, all statistical analyses were performed using the R statistical programming environment [14]. Assessment of allele frequencies were conducted using the computer programmes Haploview [15] and PLINK [16]. Allelic association statistics were calculated using the chi-squared test, with correction for multiple testing by replication in the three populations. Haplotype blocks were defined as a group of htSNPs whose upper 95% confidence bound for D' exceeded 98% with the lower bound above 70% [17] and a haplotype was examined if it occurred in more than 1% of individuals. Haplotypes were tested for association with ALS risk using the chi-squared test.

The data for angiogenin levels were assessed for the reported influence of age and sex [18]. Using data pooled from cases and controls in all three populations, angiogenin levels were regressed against age and sex and an outlier was identified and removed if its studentized residual exceeded the critical *t* statistic for the group's Bonferroni-corrected 5% significance threshold. The regression analysis was then re-iterated until no further outliers could be identified. Four Swedish plasma values and four Swedish CSF values were removed this way. The resulting linear models were used to adjust the values in the respective groups based on age and sex. The influences of genotypes across the five htSNPs were then assessed by analysis of variance (ANOVA) for each htSNP and the differences between case and control angiogenin levels for each genotype were assessed for statistical significance using the Mann-Whitney-Wilcoxon test. Finally, using data from the Swedish population, corrected plasma angiogenin levels were assessed for correlation with corrected CSF angiogenin levels in ALS patients and in controls independently.

## Results

### *ANG* SNP and haplotype association

The mean genotyping call rate across all htSNPs in the three populations was 98.4%. No htSNP deviated significantly from Hardy-Weinberg equilibrium in any study population. The results for the allelic association tests for the five htSNPs are shown in table 1. Linkage disequilibrium (LD) between htSNPs is shown in Figure S1. All five htSNPs showed association with risk for ALS in the Irish study group, with one htSNP, rs17114699, replicating in the Swedish population (p*_Irish_*  = 0.03; p*_Swedish_*  = 0.001). No htSNP showed association in the Polish population. A haplotype block was identified in all three populations, incorporating SNPs rs9322855, rs8004382 and rs4470055. The AAG and CGA haplotypes at these three SNPs associated with ALS in the Irish data, while the AGG haplotype showed strong association with ALS in the Swedish data (table 2).

**Table 2:** Haplotype frequencies and association statistics in the three populations.

Haplotype	IRELAND			SWEDEN			POLAND		
	Freq (ALS;ctrl)	p	Permuted p	Freq (ALS;ctrl)	p	Permuted p	Freq (ALS;ctrl)	p	Permuted p
AAG	0.45; 0.53	0.024*	0.13	0.46; 0.47	0.64	0.99	0.453; 0.474	0.4498	0.95
CGA	0.29; 0.22	0.023*	0.12	0.25; 0.25	0.68	1.00	0.256; 0.279	0.3627	0.90
CGG	0.18; 0.16	0.43	0.94	0.18; 0.24	0.027	0.16	0.193; 0.172	0.3424	0.88
AGG	0.07; 0.08	0.55	0.98	0.097; 0.045	<0.0001*	0.0006*	0.099; 0.075	0.1311	0.51

*Significant p-value.

### Plasma, serum and CSF angiogenin levels

Age and sex both had a significant effect on angiogenin levels in plasma/serum and in CSF (P(>|t|) <0.0001 for all covariates). Using data pooled from the three populations and after correcting for age and sex, angiogenin levels were significantly lower in ALS patients than in controls in plasma/serum (mean ± SD  = 438.2±112.2 ng/ml for the ALS group and 467.6±105.4 ng/ml for controls; p = 0.001, Mann-Whitney-Wilcoxon test) and in CSF (mean ± SD  = 5.582±1.754 ng/ml for the ALS group and 6.197±1.987 ng/ml for controls; p = 0.01, Mann-Whitney-Wilcoxon test). Angiogenin levels did not differ significantly depending on whether they were measured from serum or plasma (p = 0.93; Figure S2). There was a significant positive correlation (p<0.0001, Pearson product-moment correlation) between corrected CSF angiogenin levels and corrected plasma angiogenin levels in controls, whereas in ALS patients (p = 0.21) the observed correlation was attenuated (figure 3; r^2^ *_control_*  = 0.13, r^2^ *_ALS_*  = 0.011).

**Figure 2:**
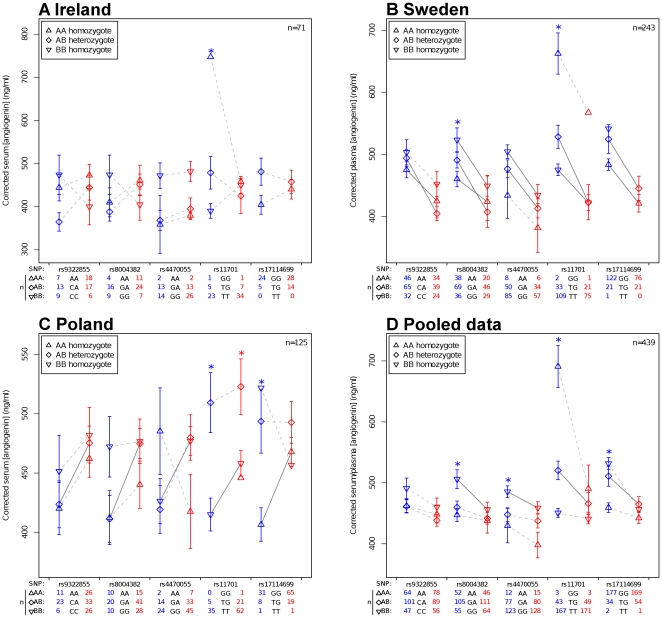
Mean corrected serum or plasma angiogenin concentrations as a function of *ANG* htSNP genotype. ALS patients are shown in red and controls are shown in blue. Significant differences between ALS patients and controls are denoted by solid lines and significant F-statistics within groups are denoted by asterisks. Error bars are standard error of the mean. Numbers of observations for each genotype at each SNP are indicated in the table below each plot.

### Contribution of SNP genotypes to angiogenin levels

Levels varied considerably around the fitted models (multiple r^2^ *_serum/plasma_*  = 0.074; multiple r^2^ *_CSF_*  = 0.16). ANOVA was used to assess the contribution of genotype at each htSNP to the overall variance in the data and the Mann-Whitney-Wilcoxon test was used to assess the differences between corrected plasma/serum levels in ALS patients and controls for each SNP, separated by genotype. Data were analysed both as independent populations and also as a pooled dataset. The results of these tests, along with the group means, are reported in figure 2.

**Figure 3:**
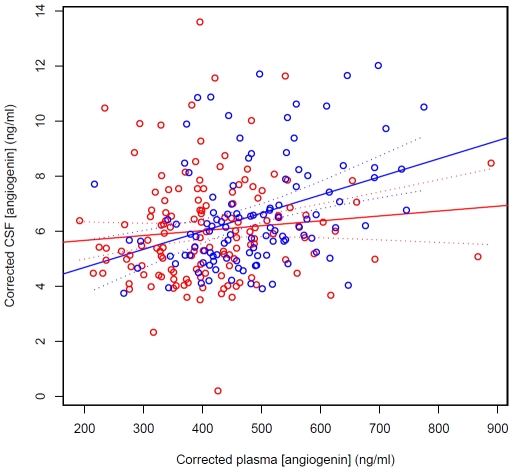
Correlation of CSF angiogenin levels with serum angiogenin levels in the Swedish population. ALS patients are shown in red and controls are shown in blue. Dashed lines indicate 95% confidence intervals of the regression lines. r^2^ values are: controls, 0.13; ALS, 0.011.

In the large Swedish dataset, an allele dose-dependent regulation of plasma angiogenin was readily observable for all SNPs in controls and perturbation of this pattern was seen in ALS patients at SNPs rs8004382 and rs9322855. These findings are reflected in the pooled dataset. Only at SNP rs11701 was a significant contribution of genotype to the variance in controls observable in all three populations; however, in the pooled dataset genotypes at every SNP except rs9322855 were shown to contribute significantly to variance in controls. No SNP contributed significantly to variance in ALS patients in the pooled data, however this was observed at rs11701 in the Polish dataset.

## Discussion

This study confirms the previously observed association between *ANG* variants and ALS in the Irish population [1], with 5 htSNPs across the *ANG* gene showing association with ALS. One htSNP, rs1711699, replicated in the Swedish cohort, showing strong association with ALS risk (p = 0.001). We have also demonstrated that two *ANG* haplotypes in the Irish and one in the Swedish associate with ALS, adding strength to the argument that *ANG* is implicated in the pathogenesis of sporadic ALS. Although replication in the Swedish population increases our confidence in the Irish findings, no htSNP or haplotype associated with ALS in the Polish population. Similarly, in a recent screen for replication of findings from the Irish genome-wide association study for ALS risk [19] using a Polish dataset, the results were surprisingly uninformative [20]. The failure to replicate in the Polish population may reflect true population based differences, as has been recently demonstrated both in population genetics [21] and with respect to other risk genes in ALS [20]. Together, these findings suggest that the complex genetics of ALS differ between the Polish, Swedish and Irish populations.

Figure 2 (notably parts a and c) demonstrates the need for large datasets when analysing data that vary so substantially by chance. However, using pooled data we have shown that contribution of SNP genotypes to variance in angiogenin levels in serum is evident in neurologically normal individuals, and that this is abolished in ALS. In controls, this contribution of genotype to variance is allele dose-dependent. SNP genotypes at rs11701 were observed to contribute to variance in ALS patients in the Polish; this finding is consistent with the observation that no *ANG* SNP or haplotype associated with ALS in the Polish.

Using the current Irish dataset, we were unable to replicate our previous finding that serum angiogenin levels are higher in ALS patients compared to controls [8]. Using our current data pooled with Swedish and Polish populations, we have shown that angiogenin levels are in fact significantly lower in ALS patients than in neurologically normal controls (p<0.001). Moreover, sub-categorisation of ALS patients and controls by SNP genotypes maintains the significance of the case-control differences in angiogenin levels (figure 2).

The differences between the current data and our previous findings most likely relate to differences in our statistical management of the dataset. In the original study we considered the effects of covariates (age, sex) in ALS patients and controls independently. In the current analysis, we more correctly assumed that angiogenin levels in ALS patients would follow the same patterns based on age and sex as those observed in controls. Thus serum angiogenin levels were initially regressed against age and sex using combined data from cases and controls. This methodology permits a more robust estimate of the influence of age and sex on angiogenin levels, as it uses approximately twice as many values (541 values) as would be used if considering cases and controls separately. Indeed, re-analysis of the current dataset using our previous methodology yielded a significantly higher mean corrected angiogenin level in cases than in controls (p<0.0001); we now consider this to be a less accurate interpretation of the available data.

In neurologically normal controls, plasma angiogenin concentration predicts CSF angiogenin concentration (p<0.0001, figure 3). We have shown that this correlation is lost in ALS patients (p = 0.21 for patients), which may suggest a tissue-specific dysregulation of angiogenin expression in ALS. This could be due to a number of factors, including perturbation of angiogenin transport in ALS, however an interesting possibility could be micro RNA (miRNA) regulation of angiogenin expression. Altered miRNA regulation of progranulin has been reported recently in frontotemporal dementia [22]. As progranulin is functionally similar to angiogenin, and frontotemporal dementia is biologically related to ALS [23], a similar form of altered regulation of angiogenin may apply in ALS. A search the EBI's miRBase Sequence Database [24] using the online Microcosm web application reveals 19 potential miRNA binding sites in the *ANG* gene for 24 human miRNAs, some of which may be preferentially expressed in the central nervous system [25]. This suggests a possible mechanism for our observed tissue-specific differences indicating that further investigation of miRNA regulation of angiogenin is warranted.

In summary, we have confirmed that *ANG* variants associate with ALS in the Irish and also in the Swedish. We have also shown that angiogenin expression is modulated by genetic variation across the *ANG* gene in an allele-dose dependent manner, and that this regulation is disrupted in ALS patients. The finding that plasma angiogenin level does not predict CSF angiogenin level in ALS patients suggests a tissue-specific regulation of angiogenin levels that may be determined by genetic variation [18]. In light of these findings, further investigation of angiogenin regulation in ALS is justified.

## Supporting Information

Figure S1 **Linkage disequilibrium between the five** ***ANG*** **SNPs in the three populations.** (PDF)

Figure S2 **Boxplot comparing angiogenin levels measured in plasma from Swedish individuals (n = 320) and serum from Irish and Polish individuals (n = 220).** The difference between the two datasets is not statistically significant (p = 0.93). (PDF)
