# Supplementary figures and images for: Angiogenin Levels and ANG Genotypes: Dysregulation in Amyotrophic Lateral Sclerosis
Source: PLoS One. 2010 Nov 10;5(11):e15402. doi: 10.1371/journal.pone.0015402 (PMC2978104; doi:10.1371/journal.pone.0015402)

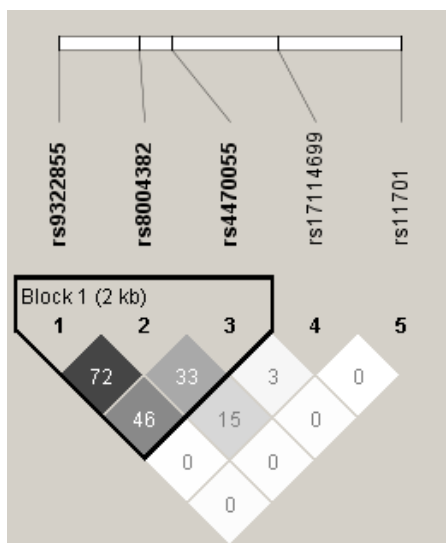

(a) Ireland  $r^2$

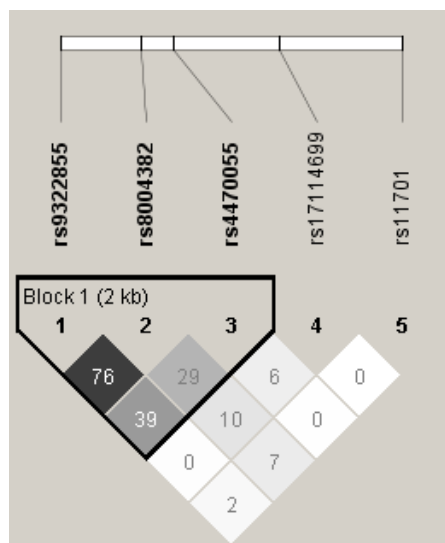

(b) Sweden  $r^2$

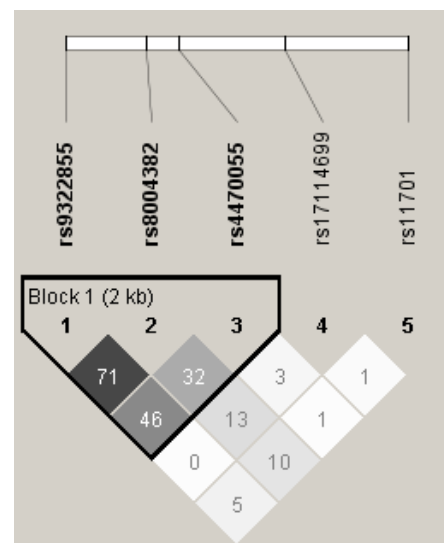

(c) Poland  $r^2$

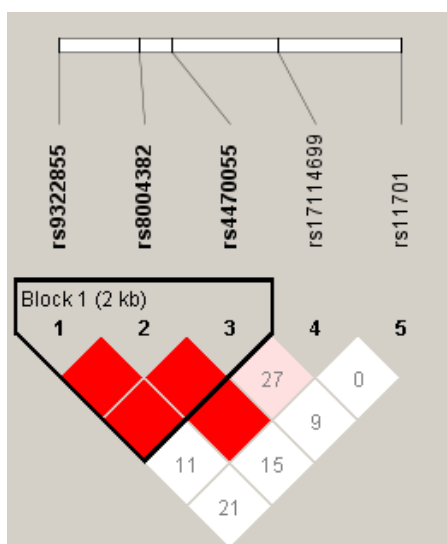

(d) Ireland  $D'$

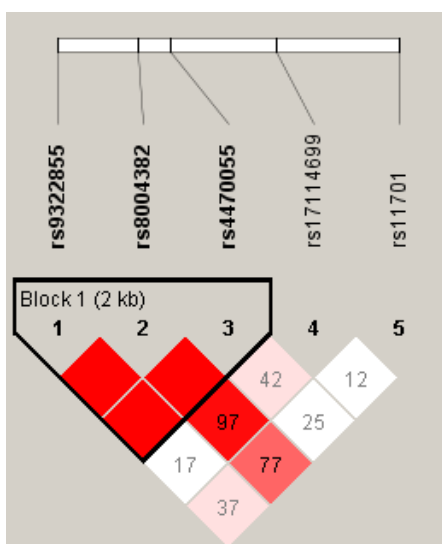

(e) Sweden  $D'$

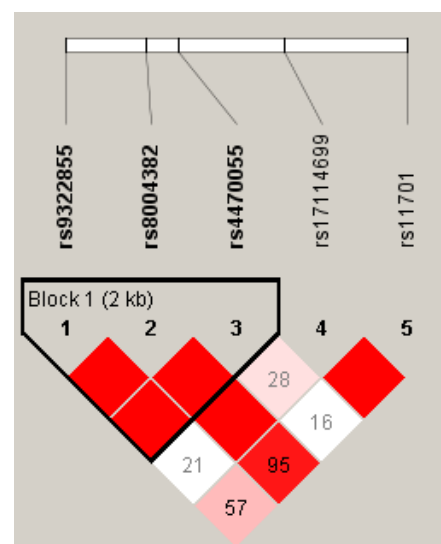

(f) Poland  $D'$

Supplement: Figure S1 — Linkage disequilibrium between the five ANG SNPs in the three populations. (PDF) [file pone.0015402.s001.pdf]

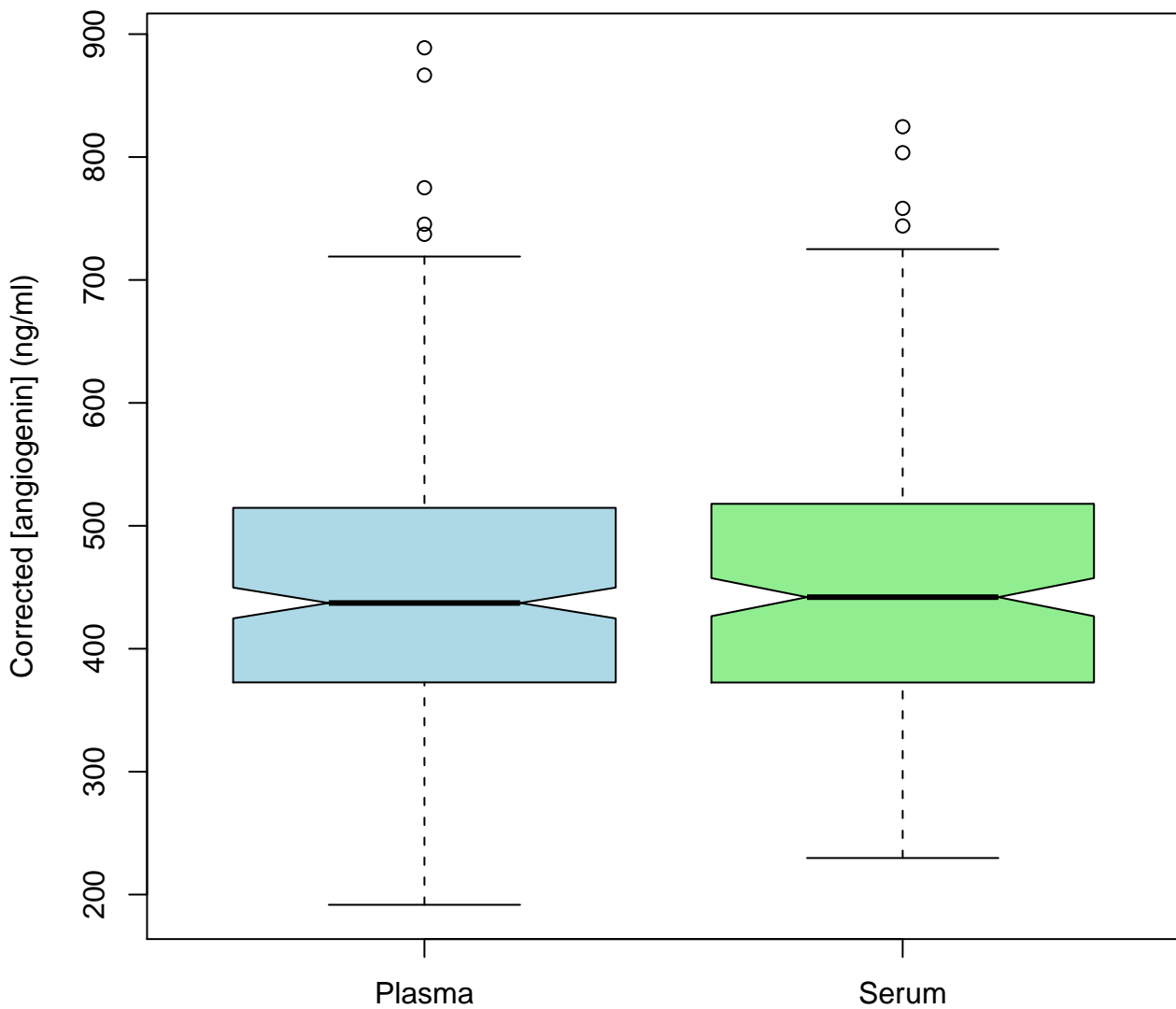

Supplement: Figure S2 — Boxplot comparing angiogenin levels measured in plasma from Swedish individuals (n = 320) and serum from Irish and Polish individuals (n = 220). The difference between the two datasets is not statistically significant (p = 0.93). (PDF) [file pone.0015402.s002.pdf]
